# Borders of STN determined by MRI versus the electrophysiological STN. A comparison using intraoperative CT

**DOI:** 10.1007/s00701-017-3432-5

**Published:** 2017-12-23

**Authors:** Sander Bus, Pepijn van den Munckhof, Maarten Bot, Gian Pal, Bichun Ouyang, Sepehr Sani, Leo Verhagen Metman

**Affiliations:** 1 Departments of Neurological Sciences, Rush University Medical Center, Chicago, IL USA; 2 Department of Neurology, Onze Lieve Vrouwe Gasthuis (OLVG), 1091 AC Amsterdam, The Netherlands; 3 Department of Neurosurgery, Academic Medical Center, Amsterdam, The Netherlands; 4 Departments of Neurosurgery, Rush University Medical Center, Chicago, IL USA

**Keywords:** Deep brain stimulation, Subthalamic nucleus, Microelectrode recording, Susceptibility weighted imaging, T2-weighted imaging, Intraoperative computed tomography

## Abstract

**Background:**

It is unclear which magnetic resonance imaging (MRI) sequence most accurately corresponds with the electrophysiological subthalamic nucleus (STN) obtained during microelectrode recording (MER, MER-STN). CT/MRI fusion allows for comparison between MER-STN and the STN visualized on preoperative MRI (MRI-STN).

**Objective:**

To compare dorsal and ventral STN borders as seen on 3-Tesla T2-weighted (T2) and susceptibility weighted images (SWI) with electrophysiological STN borders in deep brain stimulation (DBS) for Parkinson’s disease (PD).

**Methods:**

Intraoperative CT (iCT) was performed after each MER track. iCT images were merged with preoperative images using planning software. Dorsal and ventral borders of each track were determined and compared to MRI-STN borders. Differences between borders were calculated.

**Results:**

A total of 125 tracks were evaluated in 45 patients. MER-STN started and ended more dorsally than respective dorsal and ventral MRI-STN borders. For dorsal borders, differences were 1.9 ± 1.4 mm (T2) and 2.5 ± 1.8 mm (SWI). For ventral borders, differences were 1.9 ± 1.6 mm (T2) and 2.1 ± 1.8 mm (SWI).

**Conclusions:**

Discrepancies were found comparing borders on T2 and SWI to the electrophysiological STN. The largest border differences were found using SWI. Border differences were considerably larger than errors associated with iCT and fusion techniques. A cautious approach should be taken when relying solely on MR imaging for delineation of both clinically relevant STN borders.

## Introduction

Deep brain stimulation (DBS) of the subthalamic nucleus (STN) is an established and effective neurosurgical treatment for reducing motor symptoms in Parkinson’s disease (PD) [8, 26, 34]. In order to maximize therapeutic benefit while minimizing side effects, accurate positioning of DBS electrodes is crucial [1, 10, 27]. In most centers, identification of the optimal clinical target is performed using a combination of preoperative magnetic resonance images (MRI), intraoperative microelectrode recording (MER), and/or test stimulation [13, 21]. Improved visualization of STN on MRI has facilitated direct targeting of this nucleus [12]. Several groups are currently omitting MER and test stimulation and rely solely on MRI visualization of the STN for lead placement [11, 15].

T2-weighted MRI is most widely used for STN visualization, which displays the nucleus as a hypointense area anterolateral to the red nucleus [9]. However, it remains difficult to distinguish STN from other (also hypointense) adjacent structures like the ventromedial located substantia nigra (SN) and anterodorsal pallidofugal fiber pathways [9, 14]. Susceptibility weighted imaging (SWI) has gained interest for DBS surgery because of its distinct visualization of iron-rich structures like the STN, its reported high level of contrast-to-noise ratio, and clear depiction of (superficial) veins [20, 22, 25, 29, 31]. However, a recent study has shown that STN representation on 1.5-Tesla (T) SWI showed less correspondence with lateral electrophysiological STN borders than conventional T2-weighted imaging [5]. This is considered a disadvantage when targeting the dorsolateral sensorimotor part of the STN, the preferred target in STN DBS [5, 28]. If centers are to rely on direct targeting alone, it is critical that preoperative MR imaging accurately reflects the target. How high-field T2 and SWI correspond with electrophysiological STN borders has not been extensively studied.

In the current study, we compared dorsal and ventral STN border representation on 3-T SWI and T2-weighted MRI to the MER defined electrophysiological STN borders using intraoperative CT.

## Methods

### Patients

This retrospective study included patients who underwent MER-guided DBS of the STN for idiopathic PD between January 2014 and October 2016 at our institution. Information was collected from all patients with an available set of preoperative 3-T T1-weighted, T2-weighted and SWI scans with at least one intraoperative CT scan at target depth. All patients fit the general criteria for deep brain stimulation surgery as determined by an interdisciplinary team consisting of a movement disorder neurologist, a DBS neurosurgeon, and neuropsychologist.

### Imaging parameters

Preoperative MR images were obtained using a Magnetom Verio syngo MR B19 scanner (Siemens, Munich, Germany). Patients were awake during MR imaging. For T2-weighted 3-T images the parameters were as follows: repetition time, 5000 ms; echo time, 70 ms; slice thickness 2 mm; voxel size 0.7 × 0.7 × 2.0 mm; and acquisition time 8:47 min. For 3-T SWI images: repetition time, 30 ms; echo time, 20 ms; slice thickness 1 mm; voxel size 0.5 × 0.5 × 1.0 mm; and acquisition time 11:40 min. For T1-weighted 3-T images: repetition time, 1900 ms; echo time, 2.9 ms; slice thickness 1.2 mm; voxel size 1.0 × 1.0 × 1.2 mm; and acquisition time 7:07 min. Preoperative stereotactic CT parameters were as follows: kV 120; reference mAs was 390 and dose modulation was used; tube rotation 1; slice thickness 1 mm; pitch 0.55; scan length from skull base to vertex. Intraoperative CT parameters were as follows: field of view 20 cm; scan range 15 cm; total slices, 192; slice thickness 0.78 mm; acquisition time 13 s.

### Surgical procedure

Preoperative MR images, generally performed 1–2 weeks prior to surgery, were coregistered with stereotactic frame-based CT images using a Stealthstation S7 (Medtronic, Minneapolis, MN, USA) equipped with planning software (Framelink 5.1, Medtronic, Minneapolis, MN, USA). Three-dimensional volumetric T1-weighted scans were used to establish the anterior commissure (AC), posterior commissure (PC), and three midline points.

Patients underwent electrode implantation following MER-guided DBS of the STN under local anesthesia using a frame-based stereotactic approach (Leksell frame, Elekta, Stockholm, Sweden). Preoperative MR images were fused with the stereotactic frame-based CT scan and intraoperative CT images using the planning software. The target in the dorsolateral STN was acquired on axial SWI images by choosing the midpoint between the lateral and medial STN borders in line with the anterior border of the red nucleus (RN) where the RN is at its widest diameter. Surgery was started on the left side in bilateral cases, with patients in reclined position with head of the bed at 30°. Fibrin glue was applied to prevent excessive cerebrospinal fluid loss following burr hole placement and positioning of the microelectrode holder prior to MER. Single track MER through the central channel of a multielectrode holder (Bengun, Alpha Omega, Nazareth, Israel) was performed for intraoperative electrophysiological refinement of the target. Test stimulation was performed following MER to assess clinical effect and side-effect profile.

iCT images were obtained using O-arm technology (Medtronic, Minneapolis, MN, USA). The O-arm gantry was positioned concentrically with the patients’ head. Positioning of the gantry in this fashion is important to visualize all cranial structures from the vertex of the skull to the skull base. This allows for accurate merging with stereotactic CT and preoperative MR images. Two preset memorized positions were used during surgery. The ring was lowered and tilted towards the feet of the patient when a scan was performed. During the procedure, the ring was positioned more vertically to allow unrestricted access for the surgical team. iCT images were obtained systematically after the initial MER track and after final DBS lead placement in each hemisphere. iCT was repeated if additional MER tracks warranted visual confirmation, for instance when conflicting data was obtained after MER and test stimulation. Merging of the preoperative images and subsequent intraoperative CT images was visually inspected by the DBS neurosurgeon (SS). If the merging was inadequate, iCT was repeated. Final leads (lead model 3389, Medtronic, Minneapolis, MN, USA) were placed in the optimal track. Pulse generators were placed in the same surgical session, or a week following implantation of the DBS leads.

### Electrophysiological defined STN (MER-STN)

MER was started 15 mm above the intended target, and advanced continuously in submillimetric increments to approximately 3 mm below target until electrophysiological STN signal was lost or substantia nigra pars reticulata was encountered. Electrophysiological STN activity was considered to be present when background noise increased and an irregular discharge pattern with occasional bursts was detected during MER. Electrophysiological recordings were obtained and interpreted by a neurologist (LV and GP) during surgery. When STN cells were encountered, the level of depth in relation to the intended target was noted and kinesthetic responses were sought. Dorsal and ventral electrophysiological borders of the STN were determined for each MER track after review of intraoperative notes, as was electrophysiological activity at target depth which was scored dichotomously as being present or absent.

### MRI defined STN (MRI-STN)

Each track was projected on T2 and SWI sequences. Dorsal and ventral borders were evaluated for each track on axial and coronal slices. Sagittal slices were not consistently used, as the quality of this reconstructed plane made it impossible to consistently visualize dorsal and ventral STN borders. Trajectories were created by selecting an entry and target point on iCT images of microelectrode and DBS lead artifacts. The tip of the microelectrode or DBS lead, which appears as a clear hyperdense artifact, was selected as the target. An entry point was selected more cranial along the artifact, at the most proximal part of the artifact. The center of the artifact was chosen on axial, coronal, and sagittal slices. The ‘trajectory view’ setting was used to refine selection of the artifact tip for both microelectrodes and DBS leads. Dorsal and ventral borders were determined and we documented their respective distances to target.

In addition, the presence or absence of STN activity at target depth was determined. The optimal resolution window was chosen for STN representation. This was done manually for each imaging sequence by adjusting the level and width sliders to acquire optimal visualization of the STN in relation to its surrounding structures. On T2-weighted imaging, this was considered the resolution by which the STN appeared as a hypointense structure located lateral to the anterior border of the (hypointense) red nucleus, with hyperintense white matter tracts surrounding it [9]. For T2 images, a level of 820 and a width range of 1080–1180 was chosen. The same method was used for SWI images. For SWI images, the STN appeared as a hypointense structure uniformly surrounded by hyperintense white matter tracts lateral to the (hypointense) red nucleus. For SWI, a level of 280 and a width of 264 was chosen. For equal comparison, these settings were used for all images. These settings were chosen after consensus by the first and last author, after reviewing the first five cases.

**Fig. 1:**
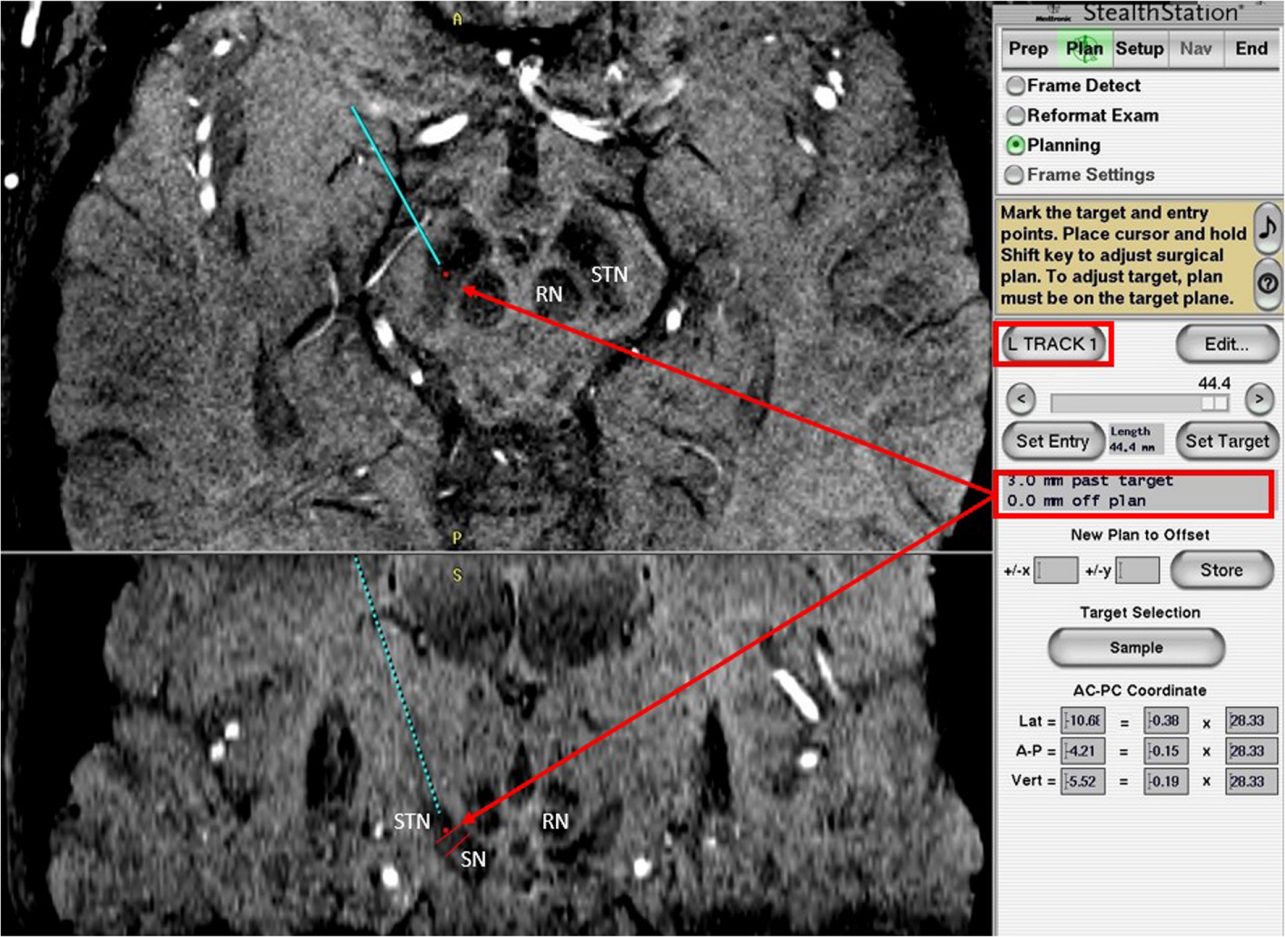
Delineation of the ventral MRI-STN border. Illustration of ventral MRI-STN border delineation along a MER track. The MER track is projected on a 3-T SWI scan. Both axial (*top*) and coronal (*bottom*) planes are visualized. The targeting dot, indicated by the *arrow*, represents the tip of the ME. The planning software is used to extrapolate the track past target depth by manually entering values into planning software, 3 mm past target in this illustration. On the axial image, the tip of the ME is inside the subthalamic nucleus (STN), on the border between the more anteromedially located substantia nigra (SN) and at the level of the anterior border of the red nucleus (RN). On the coronal image, the two parallel lines illustrate the ‘channel’ between the STN and the SN. We considered the most lateral aspect of this the ventral STN border. In this case, 3 mm past target

Borders of STN on both sequences were determined for each MER track by manually entering depth changes in relation to target depth into the planning software. Steps of 0.5 mm along the trajectory were used for border identification. Borders were defined as the value, given in mm in relation to target depth, for which the trajectory was situated inside the STN on axial and coronal images. The dorsal border was defined as the last slice where the trajectory was still in the hypointense nucleus before entering the dorsal white matter tracts. The ventral border was identified by determining the last slice on which the trajectory was located in the hypointense nucleus, without having entered the SN. The start of the SN was represented by an increased hypointense area ventromedial to the STN, when the posterolateral tail of the STN could no longer be identified on axial images. On both T2-weighted and SWI sequences, a small less hypodense area between the STN and SN was often visible on coronal images. We considered this area to be in between STN and SN. It was noted that the SN appeared more hypointense than the STN on both sequences. All trajectory borders and representation of STN at target depth were determined by two independent reviewers who were unaware of MER measurements (SB and LVM). In case of disagreement, images were reviewed again and borders were determined after consensus was reached. To prevent bias in retrospective data collection, MRI-STN borders for each track were determined before review of MER-STN borders. We compared delineation of STN defined by MER, which we took as the golden standard, with the STN delineated on both MRI sequences. Figure 1 illustrates MRI-STN border determination.

### Analysis

Differences between borders were calculated to compare track representation on both sequences to the MER-STN. Sensitivity, specificity, negative predictive value (npv) and positive predictive value (ppv) were calculated for both sequences. Accuracy of stereotactic methods was calculated by comparing intended target to the final DBS leads on iCT. Euclidian distance (ED) was calculated using the following formula:$\sqrt{\left(\Delta  {X}^2\right)+\left(\Delta  {Y}^2\right)+\left(\Delta  {Z}^2\right)}$. Statistical analysis was performed with SAS 9.1 (SAS Institute Inc., Cary, NC, USA). Data are presented as mean (± SD) and statistical significance was defined as *p* < 0.05.

## Results

### Patients and stereotactic target

A total of 62 patients underwent STN-DBS between January 2014 and October 2016. Seventeen people were excluded; in 16 cases this was due to missing iCT data and in one case due to missing intraoperative notes. A total of 88 dB electrodes were implanted in the STN of 45 patients with PD. Two patients underwent a unilateral procedure. There were 34 male and 11 female patients. Direct targeting resulted in stereotactic coordinates of 11.5 ± 1.0 mm lateral, 2.8 ± 0.91 mm posterior, 4.3 ± 1.4 mm inferior to the midcommissural point. Intraoperative CT scans were performed after reaching target depth for 125 tracks. Three SWI series were missing. Two T2 and one SWI scan were excluded because of poor image quality. MER data was missing for one patient and excluded in another patient because of calibration errors. For border comparison analysis, tracks were excluded if no STN activity was encountered during MER, or if the track was determined to miss the STN completely on MRI. A total of ten tracks (four left, six right) on SWI, and ten tracks (four left, six right) on T2 were excluded. Patient demographics are summarized in Table 1.

**Table 1:** Patient demographics

	*N* (%)
Patients	45
Male	34
Female	11
Age (range)	62 (41–75)
Total MER tracks	167
Left	87
Right	80
MER passes needed L	
1	15 (34)
2	17 (39)
3	10 (23)
4	2 (5)
Total implantations	44
MER passes needed R	
1	19 (43)
2	16 (36)
3	7 (16)
4	2 (5)
Total implantations	44
DBS lead placement L	
Central channel	13 (30)
Non-central channel	31 (70)
Total implantations	44
DBS lead placement R	
Central channel	24 (55)
Non-central channel	19 (43)
Unclear	1 (2)
Total implantations	44
DBS lead placement L + R	
Central	37 (42)
Non central	50 (57)
Unclear	1 (1)
Total implantations	88
Vector errors in mm (mean ± SD)	
Plan vs. DBS lead	2.6 (1.4)
Plan vs. first track	2.3 (1.5)
First track vs. DBS lead	3.1 (1.5)

### Microelectrode tracks

A total of 167 MER tracks were performed, 87 in the left and 80 in the right hemisphere. In 39% (34/88) of implantations only one MER pass was needed. The central channel was chosen for final electrode placement in 42% (37/88) of implantations, in 29.5% (13/44) of left-sided and 55% (24/44) of right-sided implantations. Final lead position could not be retrieved from the intraoperative notes for one the right-sided DBS lead. The DBS lead was placed in a position without electrophysiological exploration in seven left-sided and four right-sided implantations. In these cases, we decided to lower the DBS lead in a location with presumed better side effect thresholds, away from the internal capsule.

### Border representation

Dorsal and ventral borders of the MRI-STN on both sequences were compared to dorsal and ventral borders of the MER-STN. On T2 sequences, 212 borders were compared in both hemispheres of which 47.2% (100/212) borders showed typical STN activity with MER. Of dorsal borders, 67% (71/106) showed typical STN activity with MER. For ventral borders, this was 27% (29/106). For SWI, a total of 200 borders were compared, of which 40% (80/200) showed typical STN activity with MER. Of dorsal borders, 57% (57/100) showed typical STN activity. For ventral borders, this was 23% (23/100). No statistically significant differences were found between the two sequences when comparing 96 dorsal (*p* = 0.09) and ventral borders (*p* = 0.49). Table 2 illustrates border correspondence.

**Table 2:** Overview of correspondence between MRI-STN and MER-STN border representation

MRI-sequence	Border	Total *N*	*N* (%) STN +
T2	Dorsal	106	71 (66.98)
	Ventral	106	29 (27.36)
SWI	Dorsal	100	57 (57)
	Ventral	100	23 (23)
Border (*n*)	T2 *n* (%)	SWI *n* (%)	*p* value
Dorsal (*n* = 96)	64 (66.67)	56 (58.33)	0.09
Ventral (*n* = 96)	25 (26.04)	22 (22.92)	0.49

Upper table illustrates correspondence between border representation of 106 tracks on T2 and 100 tracks on SWI and electrophysiological STN recordings. Borders of both MRI sequences were determined in relation to target depth, and compared with track recordings. Borders showed either typical STN activity (STN +) or absence of STN activity. These values are presented in the last column. In the lower table, 96 tracks were compared where both a SWI and T2 sequence was available for analysis. This table shows the amount of border which corresponded with typical STN activity and directly compares both sequences. *p* values indicate no statistically significant differences between border representation on both sequences

**Fig. 2:**
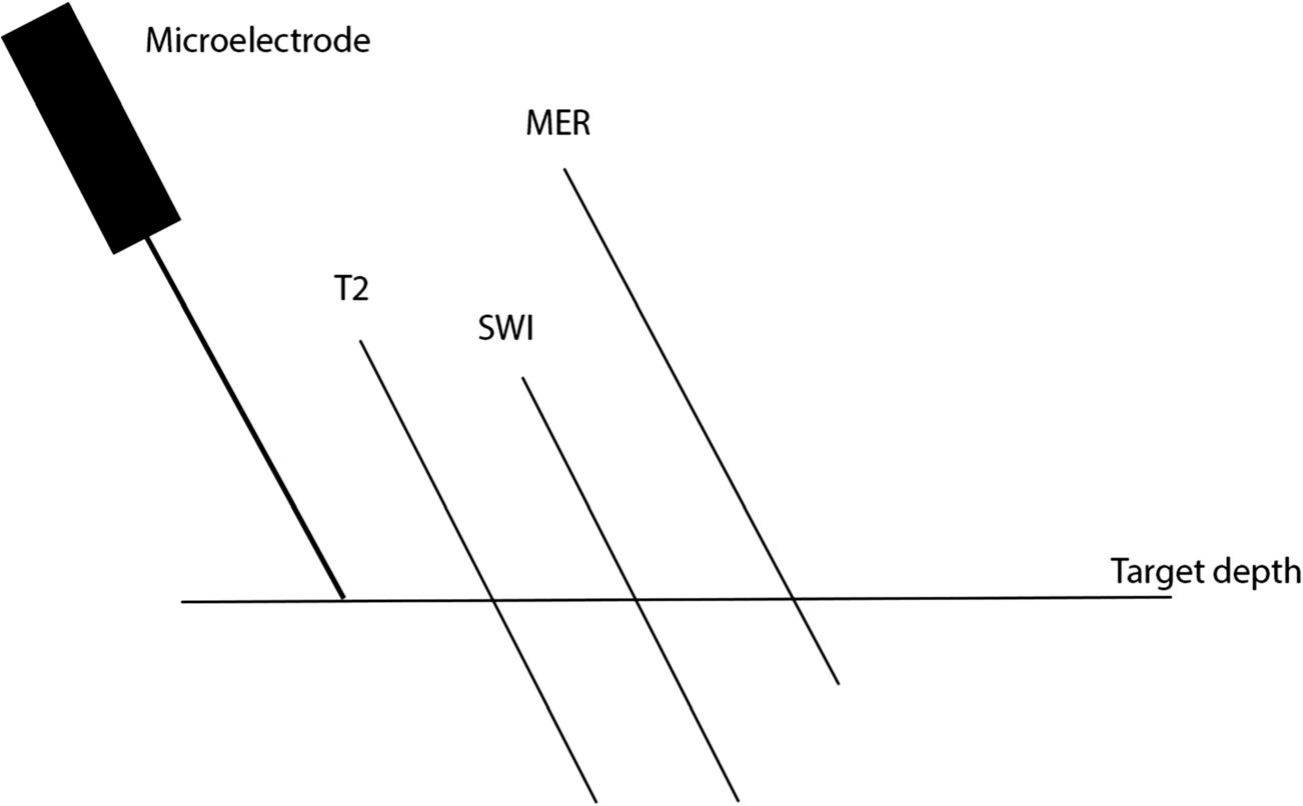
STN representation on T2, SWI, and MER along ME trajectory. This figure illustrates the representation of the MRI-STN and MER-STN along the MER track respective to each other and in relation to target depth. For reference, a microelectrode is schematically drawn next to the T2-STN, SWI-STN, and MER-STN representation along the MER track. This figure illustrates how the MER-STN starts more dorsally than the dorsal MRI-STN border and ends more dorsally than the ventral MRI-STN borders on both sequences

**Fig. 3:**
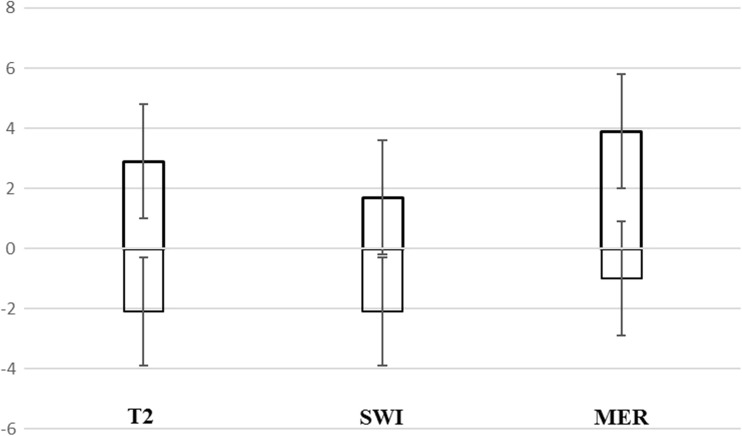
STN border representation on T2, SWI, and MER. This chart illustrates the representation of the MRI-STN and MER-STN along the MER track in relation to target depth. *Bars* illustrate the representation of the STN and its dorsal and ventral borders. The standard deviations for the mean borders are illustrated by the error lines. The *y-axis* represents millimeters from target depth (depth ‘0’), with positive values representing the dorsal aspect of the STN along the MER track and negative values the ventral aspect. MER-STN starts more dorsally than the dorsal MRI-STN border and ends more dorsally than the ventral MRI-STN borders on both sequences. T2-STN starts 2.9 ± 1.9 mm above target depth, SWI 1.7 ± 1.9 mm and MER 3.9 ± 1.9 mm. T2-STN lower borders were found 2.1 ± 1.8 below target depth, SWI 2.1 ± 1.8 mm and MER 1.0 ± 1.9 mm

MER-STN started and ended more dorsally than respective dorsal and ventral MRI-STN borders on both sequences. Distances between dorsal MER-STN and MRI-STN borders were 1.9 ± 1.4 mm (T2) and 2.5 ± 1.8 mm (SWI). Distances between ventral borders were 1.9 ± 1.6 mm (T2) and 2.1 ± 1.8 mm (SWI). Dorsal borders were identified at a mean (± SD) distance of 2.9 ± 1.9 mm above target depth for T2; 2.7 ± 1.9 mm for SWI and 3.9 ± 1.9 mm for MER. Ventral borders were identified at a mean (SD) distance of 2.1 ± 1.8 mm below target for T2; 2.1 ± 1.8 mm for SWI and 1.0 ± 1.88 mm for MER. Mean STN length was 4.7 ± 2.1 mm on T2, 4.5 ± 2.2 mm on SWI and 4.9 ± 1.7 mm utilizing MER. Figs. 2 and 3 illustrate track representation on T2, SWI and following MER.

### Target depth analysis

MER detected STN activity at target depth in 65% (80/123) of tracks. A total of 118 MER tracks were projected on T2 images of which 82.2% (97/118) were determined to be inside the STN at target depth. For SWI, 112 tracks were reviewed of which 82.1% (92/112) were determined to be inside the STN at target depth. Of tracks determined to be inside the STN at target depth, 72.2% (70/97) of tracks on T2 and 70% (64/92) on SWI showed typical STN activity at target depth. This is illustrated in more detail, along with the sensitivity, specificity, ppv, and npv in Tables 3 and 4. In a subset of MER tracks, which were chosen for DBS lead implantation, we found a sensitivity of 83% (T2) and 89% (SWI), and a specificity of 0%, as illustrated in Table 5.

**Table 3:** Overview of track representation at target depth

	T2			SWI		
Target depth	STN +	STN -	(total)	STN +	STN -	(total)
In	70	27	97	64	27	92
Out	12	9	21	9	12	21
Total	82	36	118	73	39	112

First vertical columns indicates whether the track at target depth is situated inside (In) or outside (Out) of the STN as represented on T2 and SWI sequences. STN + indicates typical electrophysiological STN activity at target depth as recorded during MER; STN - indicates an absence of typical electrophysiological STN activity at target depth as recorded during MER

**Table 4:** Measures of performance for both MRI sequences

Test parameter	T2	SWI
Sensitivity	85% (70/82)	88% (64/73)
Specificity	25% (9/36)	31% (12/39)
PPV	72% (70/97)	70% (64/92)
NPV	43% (9/21)	57% (12/21)

First vertical column lists performance measures. Sensitivity; specificity; PPV, positive predictive value; NPV, negative predictive value. Values for T2 and SWI are presented as a percentage, followed by the fraction as derived from Table 3

**Table 5:** Overview of *optimal* track representation at target depth

	T2			SWI		
Target depth	STN +	STN -	(total)	STN +	STN -	(total)
In	33	7	40	32	6	38
Out	7	0	7	4	0	4
Total	40	7	47	36	6	42

Subgroup analysis of the MER tracks which were chosen for final DBS lead implantation. We report a sensitivity of 83% (T2) and 89% (SWI) and a specificity of 0%. Positive predictive value 83% (T2) and 89% (SWI), negative predictive value was 0% for both sequences

### Stereotactic target versus final lead placement

Euclidian distance between stereotactic target and final DBS leads was 2.6 ± 1.4 mm. Distances of 0.10 ± 1.4 mm lateral, 0.90 ± 1.3 mm posterior, and 1.2 ± 1.7 mm inferior to initial stereotactic target were recorded.

## Discussion

### Border evaluation, comparing T2 and SWI

Dorsal and ventral MRI-STN borders showed a low correspondence with the MER-STN. T2 performed better in identifying both borders than SWI, although these differences did not reach statistical significance.

Ventral borders showed a remarkably low degree of correspondence with the MER-STN. Only in 29% (T2) and 23% (SWI) of tracks did the ventral border show typical STN activity. T2 and SWI signal intensity is influenced by iron content, which is known to be increased in nigral cells of patients with PD [9]. This leads to a reduced level of contrast between the STN and SN, making border delineation more difficult. This may explain the low degree of correspondence in the present study. McEvoy et al. [22] reported on the STN-SN border morphology using SWI, reviewing 28 MER tracks in seven patients. Their group found that SWI accurately estimated the STN-SN border within 1 mm of predicted depth of the electrophysiological STN-SN border in 85.7% of MER passes, concluding that SWI MRI and electrophysiological border coincide reliably. We found a mean difference of 2.1 ± 1.8 mm between ventral MER-STN and SWI-STN borders. When looking at individual tracks, 31 of 93 (33%) analyzed ventral SWI-STN borders showed differences of 1 mm or less with the ventral MER-STN border. The considerably different results between our groups may be explained by the different methods used. McEvoy et al. reconstructed MER tracks based on the DBS lead, and only reviewed the coronal plane and both the central and lateral channels in their microelectrode array. It is unclear how their group determined the location for DBS lead implantation during surgery. If the lead is placed in the track with optimal electrophysiological activity, their more favorable outcomes may, in part, be explained. MER tracks required further refinement in many of our cases and we analyzed these suboptimal tracks as well. Not consistently looking at the medial channel may also have excluded suboptimal recordings. Their group also superimposed MER tracks 1 mm apart from the implantation trajectory which is a smaller distance than our Bengun multi-electrode holder allows for. Furthermore, our study applied different methods to delineate the (ventral) STN border on SWI sequences.

Inherent imaging errors attributed to SWI, such as nonlocal susceptibility effects, may also account for some of these discrepancies. These nonlocal susceptibility effects may cause the STN to appear larger than it actually is, though it is beyond the scope of this study to determine the extent of this in both our studies [7].

We also found that the MER-STN starts and ends more dorsally than the dorsal and ventral MRI-STN border, on both sequences. Discrepancies between borders of 2.5 ± 1.8 mm (dorsal) and 2.1 ± 1.8 mm (ventral) were found for SWI, and 1.9 ± 1.4 mm (dorsal) and 1.9 ± 1.6 mm (ventral) on T2 when compared to MER-STN. Hamani et al. [14] also noted a tendency for discrepancies to occur with the electrophysiological STN appearing more dorsally than the dorsal MRI-STN border. These discrepancies were on average less than 1 mm, leading the authors to conclude a high level of correspondence between dorsal and ventral borders. Our study reports larger distances between borders on both sequences than Hamani et al. The discrepancies between our results and those of Hamani et al. may be explained by methodological differences. Their use of a 1.5-T MRI may have made visualization of STN borders more difficult, a limitation the authors acknowledge. Reconstructing tracks based on the DBS lead, rather than using the visualized ME tip coordinates, also potentially introduces bias, as previously described. It should also be noted that their group only compares T2 MRI to the electrophysiological STN.

Our results suggest that delineation of both dorsal and ventral MRI-STN borders does not accurately correspond to the electrophysiological STN borders. In the case of the ventral STN border, this is extremely relevant, as the ventral border of the STN is considered the traditional depth for electrode placement [4]. Accurate representation of this border is of paramount importance, as suboptimal lead placement resulting in stimulation of the SN has been associated with a wide variety of adverse effects, including mania and mood disorders such as depression [3, 19, 30]. Clear delineation of the dorsal STN borders also has profound clinical consequences, as the dorsolateral part of the STN is considered the preferred target in STN DBS. Our results show that the MER-STN starts more dorsally than the MRI-STN. This has not, to our knowledge, been previously reported. This finding is intriguing, as one would expect brain shift, and ‘sagging’ of the brain, to move the MER-STN more caudally. Relying solely on MRI for lead placement could potentially lead to missing the electrophysiological dorsal STN, associated with the sensorimotor area of the nucleus, altogether.

### Subthalamic nucleus representation at target depth

Both T2 and SWI show a high degree of correspondence between MRI-STN and the MER-STN at target depth. We report a high degree of sensitivity, with SWI performing slightly better (88%) than T2 (85%), but a low specificity of 25% (T2) and 31% (SWI). The high level of sensitivity is in agreement with Polanski et al. who reported values of 76.6% for T2 (with a range of 23.1–88.9% depending on the DBS contact) and 81.8% for SWI images [29]. Polanski et al. report higher values for specificity, 73.3% for T2 and 90.6% for SWI. The differences between our studies may be explained by our use of intraoperative CT for track analysis. The advantage of this technique is that our group did not have to rely on reconstructed DBS leads for track analysis. Both of our studies determined final lead placement based on the combination of therapeutic effect, side-effect profile, and quality of MER tracks. With that in mind, reconstructing MER tracks based on final DBS leads may introduce bias as the quality of electrophysiological recordings along that trajectory would be better than those recorded in suboptimal MER tracks. MER tracks required refinement in many of our cases, and we analyzed these suboptimal recordings as well. To compare our findings with Polanski et al., we analyzed a subgroup of optimal tracks. These were defined as the MER tracks in which the DBS lead was lowered. In this subset, a sensitivity for T2 (83%) and SWI (89%) was found. Interestingly, this subgroup fails to identify true-negative STN at target depth in both sequences. This may be due to the relatively small number (*n* = 7 for T2 and *n* = 4 for SWI) of tracks determined to be outside of STN at target depth in this subgroup. When comparing ppv in all tracks to values reported by Polanski et al., we found higher predictive values for T2 (72 vs. 65%) and lower values for SWI (70 vs. 86%). For the npv, we found a more considerable discrepancy. SWI performed better in predicting true-MER-STN negative tracks at target depth (57%) than T2 (43%). Polanski et al. report substantially higher values for the npv: 82.5% (T2) and 87.5% (SWI). For direct targeting, however, the ppv is arguably of greater importance than the npv because of its ability to predict a positive MER signal in the targeted MRI-STN. SWI shows a better sensitivity, specificity, and npv than T2 – with a comparable ppv. Our results show a slight preference for SWI as the sequence of choice for direct anatomical targeting, in agreement with Polanski et al. [29].

### Study limitations

For MRI-STN border delineation along the MER track, a step size of 0.5 mm was chosen. Ideally, this would have been done in a continuous fashion. Smaller steps and more continuous border determination proved to be impossible due to limited resolution of MR images. In addition, it is important to acknowledge that while visualization of STN on the axial plane (‘true images’), reconstructed images were used for the coronal and sagittal images, which potentially introduces error. Errors associated with image fusion and the O-arm itself also need to be considered. Both of these errors have been reported to be less than 1 mm, in the range of 0.13 to 0.97 mm for image fusion and 0.7 mm for inherent O-arm error [2, 16]. Careful inspection of image fusion was performed for each case to limit this error, and iCT repeated if necessary. Furthermore, despite its inherent error, iCT has been validated after CT-MRI fusion and is an established and accurate method for visualizing (micro)electrodes [16, 23, 35]. In addition, these methodological errors are smaller than the border differences we identified and are therefore less likely to be the sole contributing factors. It should be noted that the O-arm, a flat panel cone beam scanner, is not a ‘true’ intraoperative CT scanner, as it uses fluoroscopy and three dimensionally reconstructs the image. The O-arm, however, is perfectly suited to visualize lead artifacts [16, 35, 36]. While we sought to use axial images primarily in assessing the MRI-STN, ultimately reconstructed coronal and sagittal images were necessary to determine the dorsal and ventral borders. Bias in border determination was limited by reviewing MRI-STN and MER-STN data by two independent reviewers and in different sessions. While we consider electrophysiological data the gold standard for final lead placement, this does not necessarily mean that the electrophysiological STN is the true representation of the STN. Brain shift during surgery is also a factor to consider. Though standard surgical precautions were taken to limit the effect this had on surgery, some degree of brain shift is unavoidable. Brain shift can alter the intracranial anatomy and influence DBS placement accuracy [17, 24]. This potentially introduces error when comparing the MER-STN, as visualized by iCT, to the MRI-STN – as the latter is represented by preoperative scans. A potential limitation in our methodology is slice thickness. A previous study, reporting on detection of MS plaques using a 1.5-T scanner and comparing textures on 1-mm and 3-mm thick slices, found that differences between the two were small enough to enable adequate texture analysis. This suggests that both slice thickness values used in the present study would provide a comparable degree of accuracy [32].

### Strengths

This is the first study comparing high-field T2 and SWI representation of the dorsal and ventral STN borders to its electrophysiological borders using iCT. The advantage of this technique is that it allows for track analysis and comparison without having to rely on reconstructed trajectories based on MER data or final DBS leads. Reconstructing MER tracks does not take targeting errors after initial brain penetration into account. The present study reports Euclidian distances of 3.1 ± 1.4 mm and 2.6 ± 1.4 mm between the first track and DBS lead and planned target and DBS lead, respectively. These Euclidian distances and subsequent trajectory refinements would not be accounted for when reconstructing tracks based on DBS leads. Reconstructed tracks based on DBS leads potentially introduces bias because the DBS lead is generally left in the track with optimal electrophysiological activity. Our study design made it possible to delineate MRI-STN borders while blinded for the MER-STN borders, reducing bias when comparing both. That is an important aspect of our analysis we have not found in previous reports in the literature.

### Relevance to the clinical practice

Recent surgical and technical advancements have led DBS groups to consider relying solely on direct anatomical targeting for final lead placement. While we found similar stereotactic accuracy as other groups using intraoperative imaging, it is worth noting that the central channel was used for final lead placement in 42% (37/88) of our cases [6, 37]. Further target refinement was needed in more than half of placements. This is higher than previous reports, which found that direct targeting using a MER guided approach resulted in the central trajectory being used for final lead placement in 60–75% of implantations [18, 29, 33, 38]. This may reflect differences between our institutions when it comes to selecting the ideal location for lead placement. Interestingly, in the left hemisphere, the central channel was chosen for placement in 30% of cases and in 55% of cases in the right hemisphere. This difference may be explained by coordinate adjustments following MER and test stimulation in the left hemisphere, and further refinements based on iCT images of the DBS lead tip in the left hemisphere. Our results are in agreement with previous reports which conclude that SWI is a promising technique for direct anatomical targeting. Both sequences have a similar positive predictive value for a typical STN signal at planned target depth, but would incorrectly predict STN activity in about 30% of the cases. The benefit of SWI over T2 is that it more reliably limits false positives, false negatives, and predicts a negative STN signal more accurately. With its distinct ability to visualize (venous) vascular structures, it also has an added benefit during trajectory planning [31]. Both imaging sequences show low correspondence with the dorsal and ventral MER-STN borders, most notably when comparing ventral borders. The ventral MRI borders exceed the ventral MER-borders. Relying solely on MRI to delineate the (ventral) STN borders may result in placing the DBS electrode too deep, potentially leading to undesirable long-term functional outcomes. Our results suggest both sequences have their limitations when it comes to delineation of dorsal and ventral STN borders. SWI showed larger discrepancies than T2 when comparing border delineation to MER-STN. This may be caused by inherent SWI error, notably nonlocal susceptibility effects associated with all gradient echo sequences. Further research is needed to determine if these results are reproducible at higher field strengths (7 T) and when image reconstruction techniques, such as quantitative susceptibility mapping (QSM), are applied [20].

## Conclusions

Both dorsal and ventral MRI-STN borders show a low degree of correspondence with the electrophysiological STN, most notably the ventral MRI-STN border. Differences between MRI and MER defined borders are larger than those attributed to errors inherent to iCT and fusion techniques. While T2 performs slightly better, our results suggest that both techniques have their limitations when delineating the iron-rich STN-SN boundary. High-field SWI shows a higher sensitivity, specificity, and negative predictive value but a lower positive predictive value than T2-weighted MR images. This study urges caution in relying solely on T2 or SWI images to determine dorsal and ventral borders of the STN. Representation of STN borders on T2 and SWI may not accurately represent the electrophysiological STN, suggesting an added value of MER during STN DBS surgery.
